# Impact of PM_2.5_ on Second Birth Intentions of China’s Floating Population in a Low Fertility Context

**DOI:** 10.3390/ijerph16214293

**Published:** 2019-11-05

**Authors:** Wei Guo, Yan Tan, Xican Yin, Zhongwei Sun

**Affiliations:** 1 Department of Social Work and Social Policy, School of Social and Behavioral Sciences, Nanjing University, Nanjing 210023, China; weiguo@nju.edu.cn; 2 The Centre for Asia-Pacific Development Studies, Nanjing University, Nanjing 210023, China; 3 Department of Geography, Environment and Population, School of Social Sciences, the University of Adelaide, Adelaide, SA 5005, Australia; yan.tan@adelaide.edu.au; 4 Department of Social Work & Social Administration, the University of Hong Kong, Pokfulam 999077, Hong Kong, China; xicanyin@hku.hk; 5 Department of Sociology, East China University of Science and Technology, Shanghai 200237, China

**Keywords:** air pollution, PM_2.5_, second birth intention, floating population, China

## Abstract

The total fertility rate of the Chinese population has declined dramatically over the last three decades. Research has substantiated the causal link between particulate matter (PM) and adverse health effects. However, the impact of PM on the birth intentions or fertility behavior of the childbearing population remains understudied. The paper analyzes the impact of PM_2.5_ concentration (a mixture of extremely small solid particles and liquid droplets found in the air) on the second birth intentions of the Chinese floating population. We used urban migrant population matching data at the prefectural level for the analysis. The unique datasets were derived from the Chinese Floating Population Dynamic Survey in 2014 administered by the National Health Commission, the National Prefecture-level City Matching Data administered by the National Bureau of Statistics of China, and the air pollution index PM_2.5_ collected by the Green Peace Organization. The results show that PM_2.5_ concentration has a negative impact on the second birth intentions of the floating population. This impact exhibits marked regional heterogeneity: the desire for a second birth across migrant groups living in south China decreases if PM_2.5_ concentration goes up, while migrants coming from, and living in, north China show strong intentions to have a second birth despite an increase in PM_2.5_ concentration in northern cities. The results have direct implications for the Chinese government at various levels to play a vital role in making and implementing environmental policies on the mitigation of smog to effectively safeguard the health of individuals and communities and potentially raise China’s fertility rate.

## 1. Introduction

The total fertility rate of the population in China has experienced a remarkable decline since the 1970s and has been well below replacement since 1990. The rate is even lower for the floating population (0.94) than for their counterparts living in rural areas (1.28) or urban destinations (1.01) [1]. The floating population refers to people who have lived away from the place where they registered household status for six months or more [2]. The floating population consists of two groups of migrants: the rural-to-urban migrants and the urban-to-urban migrants (e.g., university graduates seeking jobs in other cities). The stock of the floating population has increased over time, rising from 6.57 million in 1982 to 221 million in 2010, and further to 247 million in 2016, accounting for 0.7%, 16.5% and 18.0% of the overall national population in the corresponding year [3,4]. In 2010, 63.0% of the floating population was from rural areas—of which, 83.7% had moved from the countryside to cities [5]. The 2010 China population census data shows that over half (53.6%) of the floating population—termed the “new generation of migrants”—was born in 1980 and after, indicating that a high percentage of the floating population are at reproductive ages (15–49) [5]. A constellation of factors has caused fertility declines. These include the National Family Planning (or one-child) policy implemented over the last 35 years [6,7], rapid urbanization and socioeconomic development [8], increased female labor force participation, education, age at marriage, and contraceptive practice [9,10]. Dramatic economic development over the last four decades in China has exacted long-term environmental, health and social prices. A recent report shows that China’s environmental performance index was ranked 118th out of 178 countries, with air quality ranking at the third place from the bottom, and the average exposure level of PM_2.5_ concentration was the highest in the world in 2014 [11]. However, there is a paucity of empirical evidence for how environmental factors, especially large-scale and severe smog across many cities in China in recent years, impact on the fertility behavior or birth intentions of the childbearing age population (especially the floating population) in the country.

Research finds a significant causal link between morbidity or mortality and ambient particulate matter (PM) in air pollution [12]. PM is a mixture of extremely small solid particles and liquid droplets found in the air. Some particles include pollen, dust, sulphates, nitrates, acid aerosols, ammonium, element carbon, carbon compounds and metals [13]. Particularly, smog has a serious toxicological impact on human health, especially through influencing human respiratory and cardiovascular systems. Some adverse health conditions and deviant behaviors including depression, asthma, lung function, chronic obstructive pulmonary disease, suicidal tendencies, and increased hospital admissions and mortality have a clear causal relationship with PM in air pollution [14,15,16]. Despite no direct link found between smog and an individual’s fertility desire, medical studies have suggested that smog affects neonatal health, which may lead people at childbearing ages to postpone their fertility plan or be unwilling to reproduce [17,18]. Literature also shows that smog increases the risk of maternal miscarriage and adversely influences human fertility [19,20,21].

Severe smog has been a top environmental challenge in the Chinese society in recent years. Migrants are more prone to exposure to smog as many of them perform outdoor jobs as construction workers, manual laborers, and handicraft people [22]. Due to the lack of representative data, it has been difficult for researchers to examine how smog influences the fertility behavior or birth intentions of the childbearing age population in China. The China Migrants Dynamic Survey (CMDS) in 2014, administered by the National Health Commission, provides a unique dataset that enables us to analyze, for the first time, how smog impacts on the floating population’s fertility intentions. *Fertility intention* plays an important role in explaining contemporary fertility trends. This factor is one of the significant predictors of subsequent fertility behavior [23,24].

This paper addresses two questions. First, in what ways and to what extent does smog (measured as PM_2.5_ concentration level) impact on the second birth intentions of the Chinese floating population? Second, is there any spatial discrepancy between migrant populations living in northern and southern regions of China?

## 2. Determinants of Birth Intentions of the Chinese Floating Population

*Fertility policy*. Since the implementation of the National Family Planning (one child) policy in the early 1970s, there has been a dramatic decline in the total fertility rate (TFR) [25,26]. Since 2000, despite a slight growth (changing from 1.58 in 2001 to 1.70 in 2016), the TFR has been much lower than the replacement level, resulting in the rapid ageing of the Chinese population. To respond to the ageing population and related aged care and other social issues, in 2013, China started to implement a conditional two-child policy, permitting a family in which at least one of the married couple was the only child of their parents to have two children [27,28]. Since October 2015, China has abolished the iconic one-child policy and started to implement a universal two-child policy, under which a married couple can have two children [29]. Some studies argue that the two-child policy does not boost the TFR because many of the new generation of migrants are unwilling to have a second child. The key reasons include their disadvantaged position in the competitive labor market and high costs of raising a child in cities [30]. Low birth intentions have become a new norm of the floating population [31]. The era of policy driven childbearing behavior has gone, and the present two-child policy plays a limited role in influencing fertility behavior [32].

*Education*. Education is one of the most established socioeconomic determinants of fertility behavior or birth intentions [24]; it is inversely associated with fertility behavior [33]. Highly educated women tend to substitute child numbers with childrearing quality [34]. Since childrearing is time-intensive, an increase in wage rates induces a negative substitution effect on the demand for more children [35]. Previous studies in other developing countries emphasize that the educational composition of a population is a key determinant of a country’s TFR and that women’s fertility intentions tend to decline with educational attainment [36,37]. In the Chinese context, the migrant group holding a relatively high level of education has a strong capability to factor social environments into their fertility plans, leading to a sharp contrast between fertility intentions and actual fertility behavior [38]. The birth intentions of the childbearing age females of the floating population drop by their education level [31].

*Economic status*. This is a complex factor that reflects an individual’s occupation and income, and thus determines a family’s affordability to raise children. Many migrants work more hours than local urban workers and work in low-end jobs that local urban workers do not want to take [39]. Generally, the current economic status of migrants is much better than that in the 1980–1990s [38]. Studies show that annual household income positively affects both actual fertility and preferred fertility: the higher the economic status, the greater the concern about the quality of childrearing and the higher the unwillingness to have children [40,41]. Female workers at childbearing ages tend to have fewer children than unemployed ones because the opportunity cost of women engaging in socialized careers is higher than that of those not in the labor market [42]. The higher the occupational level and wages, the greater the job opportunity cost and consequently the lower their second birth intentions. However, as highlighted by some Chinese researchers, the longer the floating population live in urban areas and the more wages they earn, the greater inclination to form a family [43].

*Cultural environment*. People’s cultural attitudes towards birth differs according to an individual’s social culture, social class, and social resources [44]. For the floating population, the geographical environment and cultural differences between the origin and destination affect fertility intentions in different ways. Traditional culture in rural areas motivates people to prefer sons over daughters, and such a cultural mindset makes it difficult for rural migrants to integrate into urban societies [45]. There is a significant difference in gender preference [46]. Literature shows that the occupation of migrants influences their cultural inclusion in the destination [47]. For example, compared with farmers, migrants working in business and service sectors have a greater propensity to accept local urbanites’ view on fertility and thus cater to the fertility trends in the destination. Also, a friendly, accepting urban environment is associated with positive fertility behavior [48]. A comparative study of migrants’ living conditions in two megacities (Shanghai and Shenzhen) shows a stronger willingness of forming families and giving birth for migrants living in Shenzhen than in Shanghai [38]. This is because the former city has a more inclusive culture than the latter; it is more accepting of diverse linguistic and cultural diversities caused by a massive influx of migrants.

*Hukou-based social exclusion* and *floating experience*. China’s unique household registration system (*hukou*), established in 1958, has been used as a demographic mechanism for managing population mobility and as social engineering for distributing social security and social welfare schemes that disproportionately benefit urban citizens [49]. Hukou status has two related parts: residential location (cities or towns and rural residential locations like villages or state-owned farms) and socioeconomic status (agricultural or non-agricultural). It classifies Chinese citizens into two groups (agricultural/rural and non-agricultural/urban) and endows each with vastly different opportunities and socioeconomic status, producing social segregation and disparity. Migrants cannot enjoy the same social security benefits (age pension, medical care, unemployment, maternity, and work-related injury insurance), legal aid and children’s education as the local urban residents due to the constraints of the long-lasting hukou system [50]. This discriminatory hukou system affects migrants’ fertility desire. Many migrants live in an unstable state and experience multiple risks including unemployment, frequent movement, poor housing, and low income, potentially causing the fertility decline [51]. Some researchers further note that the rural–urban divide caused by the hukou system, and then hukou- or location-based social security system, has not yet been fully removed in China [7]. The hukou-based social exclusion hinders migrants’ integration into urban life and culture.

Due to the restriction of the hukou system, it is very difficult for the floating population to obtain permanent residency in the cities [50]. Therefore, unmarried migrants are more likely to choose permanent residency in cities and the migrants who have a family left behind often choose to take temporary or circular migration [51]. Frequent movements and unstable living conditions reduce their intentions and realization of fertility [52]. Research into the relationship between migration and fertility in other nations also show that migrants have lower fertility rates than non-migrants in their homelands but higher fertility rates than local residents in the destination [53]. The separation of migrant spouses may have a significant effect on the timing of childbearing. Adaptation to an urban lifestyle plays a lesser role in reorienting their fertility intentions because many migrants stay in the destination for a limited time [54].

## 3. Impact of Air Pollution on the Birth Intentions of China’s Floating Population

General literature in population and environment research shows that people’s experience in air pollution differs by their social strata [55]. Low-income people such as migrant workers are more likely to be exposed to toxic and hazardous work environments. Smog is extremely harmful to human health and has claimed tremendous loss of life and property of Chinese people. The number of deaths caused by PM_2.5_ in Beijing in 2010 was 2349, accounting for 1.9% of the total deaths in that year; the direct economic loss amounted to nearly US$18.6 billion [56]. Medical studies establish a significant correlation between air pollution and residents’ respiratory disease, cardiovascular disease, and asthma [57,58]. The health conditions for people with respiratory diseases such as asthma, emphysema, or bronchitis become even worse if they work in smog-affected situations [59]. Smog also affects the mental health of people, increasing depression and suicidal events [15].

Severe smog can lead to an increase in the death rate of the population, and removal of smog can result in population growth. In Los Angeles, US, a large-scale smog removal process from the 1970s to the beginning of the 21st century demonstrates that when the severity of the smog problem is reduced, a population (especially in relatively poor areas) grows significantly [60]. However, there is no study directly showing how smog reduces an individual’s birth intention or human reproductive behaviors. A relevant study in Barcelona identifies a significant association between a decrease in fertility rates and an increase in traffic-related air pollution (particularly for the coarse fraction of PM) [61]. Medical scientists find that smog can damage the health of newborn babies. A longitudinal study of 100 newborn babies aged till 7 years old in New York finds that children growing up in areas with heavy pollution are more likely to have distractions, anxiety disorders, and depression than their counterparts growing in areas with little pollution [62]. Using multinational data, Dadvand et al. [17] find that if pregnant women live in conditions with severe air pollution, their babies are likely to be underweight. For an increase by every 10 micrograms per cubic meter of PM_10_, the risk of underweight babies grows by 3%. Further, taking advantage of the natural experiment that occurred during the 2008 Beijing Summer Olympics, Rich et al. [18] reveal that an increase in air pollutants reduces the baby’s weight at birth. For every increase by one quartile deviation of the concentrations of PM_2.5_, CO, SO_2_, and NO_2_ in air, the neonatal birth weight would reduce by 18, 17, 23, and 34 g, respectively. Clearly, smog can influence the health and development of the fetus. Fertility is an important life domain and individuals have to make important decisions in this domain [43]. In an era when people are under heavy pressure, one of the major motivations for fertility is not to produce one more offspring to carry on the family line in industrialized economies. Therefore, environmental quality must be taken into account as an important factor in individuals’ decision-making process including fertility intentions. From this point of view, it is a rational choice for people to reduce or postpone baby plans and keep the next generation from being harmed by air pollution.

## 4. Data, Measures and Method

### 4.1. Data Sources

Based on three datasets (at different levels) sourced from three agencies, this study formulated a ‘city–migrant population matching dataset’ to answer the central question about how smog influences the second birth intentions of the Chinese floating population. The first dataset is the 2016 China Migrants Dynamic Survey (CMDS) data. The Survey has been carried out since 2009. However, it was not designed for longitudinal data collection. Rather it was designed to collect information on the living and development conditions of the floating population to enhance governmental management in providing health and family planning services to this segment of the population. The focal questions put to migrants vary by year while basic questions about demographic and socioeconomic status remain unchanged. The CMDS employed a stratified three-stage probability proportionate to size (PPS) sampling and encompassed 279 cities across all provinces (31) in mainland China. Thus, it provides a national representative dataset on a large sample of the floating population (*N* = 200,000). It was only in the 2014 survey that information on migrants’ intentions to have a second child or not was collected. The survey encompassed over 1500 counties across all provinces (31) and Xinjiang Production and Construction Corps in the mainland. This data is characterized by large sample size, the randomness of the sample, and full coverage and representativeness of the migrant population. The second dataset, sourced from the *2015 China City Statistical Yearbook* [2], contains economic and social statistical indicators for all prefectural cities in China. The third dataset contains information on air pollution index PM_2.5_ derived from the *2014 City’s PM_2.5_ Concentrations Ranking Report*, released by the Green Peace Organization (GPO) in 22 January 2015. GPO used web crawling to collect the PM_2.5_ data of over 300 prefectural-level cities from China National Environmental Monitoring Center and calculated the provincial- and prefectural-level arithmetic mean of PM_2.5_. Using the administrative division number of a city as the identifier, we matched the prefectural level data with the individual-level data, forming the prefectural city–migrant population matching data for the year 2014.

The data shows that over one in five (21.3%) of eligible respondents indicated intentions to have a second child (Table 1). This rate is slightly higher for those living in the southern provinces than those living in the northern regions. The average concentration of PM_2.5_ in the north reached 68.45 μg/m^3^, which was approximately 20% higher than that in the south (56.85 μg/m^3^). Over 90% of the respondents indicated that they are aware of the conditional second-child policy. Approximately 13% of the respondents were eligible for having a second child.

### 4.2. The Outcome Variable

In the 2014 CMDS, migrants were asked “Whether or not you intend to have a second child?”. The response to this question is the main dependent variable (*second birth intention*) in this paper. Considering marriage status and the actual age of childbearing, we classified the sample into three groups: “married”, “primary child-bearing ages (20–49 years old)”, and “having one child who is under 20 years old”. The valid sample size includes 34,391 individuals—of which, 38.5% were females and 61.5% were males. The mean age of the sample was 34 years.

### 4.3. The Key Explanatory Variable

**Figure 1:**
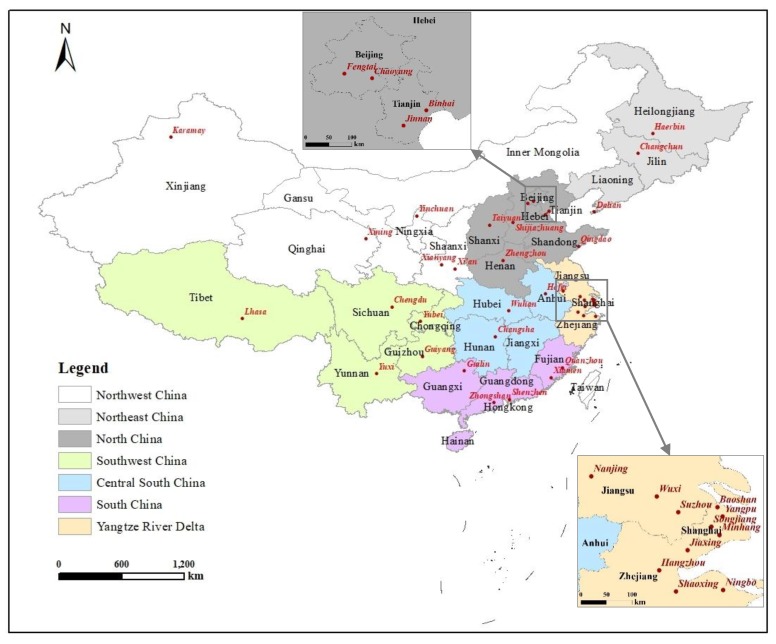
Seven subregions and 40 cities (or districts) surveyed in China. Note: Red dots on the map indicate the cities surveyed. North China consists of northwestern (Xinjiang, Gansu, Qinghai, Ningxia, Shaanxi, and Inner Mongolia), northeastern (Heilongjiang, Jilin, and Liaoning), and northern areas (Beijing, Tianjin, Hebei, Shandong, Shanxi, and Henan). South China includes southwestern (Chongqing, Sichuan, Guizhou, Yunnan, and Tibet), central–south (Anhui, Hubei, Jiangxi, and Hunan), and southern areas (Fujian, Guangdong, Guangxi, and Hainan) and the Yangtze River Delta (Jiangsu, Shanghai, and Zhejiang).

The core explanatory variable in this study is PM_2.5_ concentration. In general, air pollution is more severe in northern regions than in the southern regions of the country [63]. The most polluted areas encompass the Beijing–Tianjin–Hebei region and its neighbouring provinces including Shanxi and Shaanxi (Figure 1). These areas are the largest concentrations of China’s heavy industries, that is, coal mining, iron and steel, metallurgy, and chemical industries [64]. These areas are drought-prone and have relatively low rates of vegetation coverage. The second highly polluted areas in China include the provinces of Liaoning, Shandong, Jiangsu, and Henan. These areas are densely populated and host massive manufacturing industries emitting hazardous pollutants. The third highly polluted areas are located in the southwestern region centered in the provinces of Sichuan and Chongqing. Due to their geographical location, i.e., situated in Sichuan Basin and surrounded by highlands in west and south and mountains in north and east, tremendous pollutants generated by coal, steel, petroleum processing and other chemical industries in the region cannot be with atmospheric evacuation. Accordingly, we hypothesize that the impact of smog on birth intentions exhibits a geographical discrepancy between the north and south parts of China.

### 4.4. Control Variables

Our regression models considered a number of demographic and socioeconomic factors as control variables. These include individual-level variables (sex, hukou status, age, education, income, and social status); fertility policy; sex and age of the first child; contextual variables at the prefectural city level, e.g., gross domestic product (GDP), per capita GDP, population density, and the share of secondary industry. Controlling economic and industrial variables can avoid the interference of contextual factors at the city (macro) level on the estimation.

### 4.5. Method

Considering the sampling nature of the floating population survey, we used a weighted regression method for data analysis. The outcome variable of this research is second birth intention, i.e., whether or not migrants intend to have a second child under the premise of already having a child. This is a dichotomous variable (1 = ‘yes’, 0 = ‘no’), and so we used a Probit model to examine the relationship. Greene [65] (pp. 772–775) provided the detailed econometric specification for Probit models. In the Probit model, the inverse standard normal distribution of the probability is modeled as a linear combination of the predictors. We also tested the robustness of the results using a subsample that includes responses from migrants in 40 cities/districts in the 2014 survey (see Figure 1). These 40 cities/districts absorbed the largest floating populations and thus can be used as a representative subsample of the floating population survey to infer the situation of the entire country. To examine the individual differences in the impact of PM_2.5_ on second birth intentions, we performed the regression with interaction terms between smog and selected individual variables. To further examine any regional difference in the impact of PM_2.5_ on second birth intentions, we performed separate regressions for four categories of the floating population: “living in the south and from the south”, “living in the south but from the north”, “living in the north but from the south”, and “living in the north and from the north”. These four categories were classified in terms of the origin and current residency of migrants.

## 5. Results

Table 2, Table 3, Table 4, Table 5 and Table 6 present the results. Overall the models were a good fit as suggested by the significant Wald Chi-square statistics. The prediction accuracy of the models was quite satisfactory, being within 76% and 82% of the correct prediction for the impact models. The models did not suffer from serious multicollinearity (Mean Variance Inflation Factor/VIF = 1.61) and the robust standard errors were used to control for any heteroskedasticity.

### 5.1. Impact of PM_2.5_ on Second Birth Intentions: Reducing Intentions

A central finding drawn from Models 1–4 (Table 2) is that an increase in PM_2.5_ concentration would reduce the second birth intentions of the floating population. Model 1, shown in the first column of Table 2, considered only one independent variable, PM_2.5_. This factor has a negative association with migrants’ second birth intentions. For every one-unit increase (10 μg/m^3^) in PM_2.5_ concentration, the rate of second birth intentions of the population under study would decrease by 6.4% (*p* < 0.01), assuming other factors remain constant. After controlling for individual, institutional (policy)and contextual variables, there are very few changes in the regression coefficients and standard errors across Models 2, 3, and 4.

As expected, these results of the subsample, as presented in Model 5 of Table 2, are consistent with the results of Model 4 that used the complete sample.

Another interesting finding, as shown in Models 2–4, is that women’s second birth intention is significantly lower than that of men. When age increases, people’s second birth intentions decline. This is especially the case for the group who hold urban (or non-agricultural) hukou status, and also for the males. These findings are consistent with the findings of previous research on fertility desire and fertility behavior [66,67]. Moreover, the higher the education level, the lower the second birth intention. The rural household registers have higher second birth intentions than those urban counterparts. In terms of the first child’s sex, if a couple already has a son, their second birth intention is significantly lower than those who have a daughter. This suggests that boy preference still influences the reproductive ideology of some migrants. This result supports the findings of other researchers [31]. Employers and self-employed workers are more willing to have a second child than wage-earning migrants. Importantly, the commencement of the conditional two-child policy in 2013 exhibits a significant and substantial effect on people’s intentions to have a second child. The group who are eligible to have a second child has a much higher propensity (by 57.8%) of reproductive intentions than their counterparts who were ineligible in 2014. As the first child grows older, people’s intentions to have a second child decline. A couple of contextual factors at the city level have a significant association with second birth intentions (Model 4). Migrants residing in relatively developed and rich cities (measured as per capita GDP) have fewer intentions to have a second child than those living in less developed cities. This result is consistent with the literature [67].

### 5.2. Impact of PM_2.5_ on Second Birth Intentions: Individual Differences

Using Model 4 in Table 2 as the baseline model, we conducted a further analysis of the interactions between smog and selected individual variables: gender, educational level, hukou status, income, and sex of the first child. Such analysis provides nuanced understanding of individuals’ sensitivity to smog. Interestingly, the interaction terms between PM_2.5_ concentration and these individual variables are not statistically significant across Models 6–12 presented in Table 3. This result indicates no significant difference between diverse migrant groups differentiated by important characteristics when they experience smog. However, PM_2.5_ concentration remains to be a significant force driving the declines in second-birth intentions.

### 5.3. Impact of PM_2.5_ on Second Birth Intention: Regional Differences

The regressions were performed separately for the four migrant categories. People living in, and coming from, different regions have different levels of resilience to smog. For the group *living in the south*, regardless of their hometowns, their second birth intentions significantly decline with the increase in PM_2.5_ concentrations (Models 13 and 14 in Table 4). However, the duration of migration might be an important factor influencing the sensitivity to PM_2.5_ concentrations of the category of people *living in the south but originating from the north*. Although this conjecture cannot be confirmed from the regression results here, with the increase in the duration of stay, increased adaptive capacity to local climate and change in living habits and social-cultural norms would be affected more or less by local urbanites. Therefore, for this category of migrants, the way by which smog influences their fertility desire cannot be simply generalized. For migrants *living in the north and originating from the south*, their second birth intentions are not significantly affected by smog (Model 15 in Table 4). If their origin is in the north, their second birth intentions would increase accordingly when PM_2.5_ concentration goes up (Model 16 in Table 4). Clearly, *migrants living in the south* are more sensitive to smog when it occurs as there is relatively light air pollution in the south. The northerners have a greater resilience to smog, probably because they have become customized to smog and see it as a normal phenomenon in their lives.

To look into this issue in more detail, we divided China into seven subregions, and then conducted separate regression analyses of the microdata on the floating populations in 20 major provinces (Table 5 and Table 6). The results show that the regression coefficient of PM_2.5_ concentration is positive only in north China. Especially for migrants in Hebei province, the higher the PM_2.5_ concentration, the greater their second birth intentions. This is a puzzling phenomenon. Monitored data shows that smog and air pollution in Hebei has been the top area in China in recent years. The floating population there is dominated by intra-provincial rural migrants, and thus their resilience to smog is relatively high. Many cities there (e.g., Handan, Shijiazhuang) have high PM_2.5_ concentrations due to massive heavy industries. These cities absorb young male workers who generally have strong fertility intentions. Migrants there also have a relatively high income, so they can usually afford the cost of raising children, and then births increase.

## 6. Discussion

**Figure 2:**
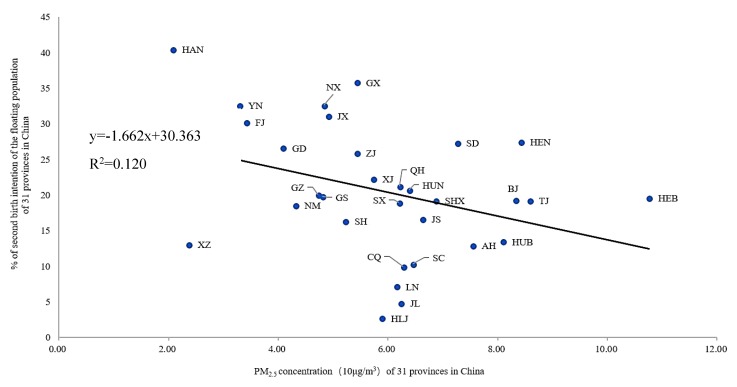
Predicted change in the probability of the second birth intentions of the floating population under different PM_2.5_ concentration conditions.

Both the fertility and natural population growth rates in China have remained at a decline over the past 20 years. Differing from previous research on the declining fertility rate of the Chinese population, which addressed the effects of demographic factors (especially age and education), Family Planning policy and rapid economic development, this study provides an environmental lens to understand the probable demographic consequence of smog risks. The findings of the study fill in part of the knowledge gap by demonstrating that the prevalence of smog significantly reduces intentions to have a second child across the Chinese floating population, and that the impact of smog on birth intentions exhibits a clear geographical discrepancy between north and south regions of China. A simple linear fit of PM_2.5_ concentration and second birth intentions indicates a significant negative but weak correlation between these two variables (with a coefficient of −0.064, at the 0.001 significance level) (Figure 2). Not surprisingly, air pollution in the provinces of Hebei (HEB), Tianjin (TJ), Beijing (BJ), Henan (HEN), Hubei (HUB), Shandong (SD), Anhui (AH), Shaanxi (SHX), Shanxi (SX), Jiangsu (JS), Sichuan (SC), and Chongqing (CQ) is more serious than in other provinces. At the prefectural city level, affected cities are mainly distributed in the north, northwest, central, and southwest regions of the country, which are dominated by heavy industries and arid or semi-arid climate. Overall, there are higher percentages of migrants with have second birth intentions in southern provinces (e.g., Hainan (HAN), Yunnan (YN), Fujian (FJ), Jiangxi (JX), Henan (HEN), Zhejiang (ZJ), Shandong (SD) and Guangdong (GD)) than there are of their counterparts living in northern regions. Smog can reduce fertility desire in at least two ways. Firstly, existing research has shown that if pregnant women are exposed to smog for a long time, it will damage the physical and mental development of the fetus or the newborn, and this effect will last until the childhood phase [17]. For the sake of the health of newborn babies, women at childbearing ages usually reduce or postpone their fertility intentions. Secondly, smog affects the physical and mental health status of child-bearing women [68,69]. Long-term exposure to smog causes not only a variety of respiratory diseases, but also mental health problems such as anxiety, depression and stress, thereby reducing their fertility intentions [14]. In the Chinese context, some floating populations in the northern areas (e.g., Hebei) have shown strong resilience to smog and have strong intentions to have a second child even with the increased frequency and severity of smog. This phenomenon is complex and will need further quantitative and/or qualitative research in the future to fully explain it.

Since the 18th National Party Congress in 2012, the Chinese central government has made great efforts to reduce air pollution. Recent developments in environmental policy and programs include setting up strict rules to control motor vehicle pollution, coal-fired pollution, key industries emitting massive pollutants, and fugitive dust pollution, and to increase ecological rehabilitation and apply new technologies to combat smog. The implementations of these programs have achieved certain plausible outcomes [63]. However, large-scale and severe smog still frequently occurs across vast parts of the country in the autumn and winter seasons. Continuing to combat air pollution is an important environmental policy to protect the health of urban and rural residents, potentially enhancing the fertility rate of the Chinese population.

## 7. Conclusions and Limitations

Coupled with rapid urbanization, socioeconomic development and heightened air pollution in China, dramatic fertility decline has occurred since the mid-1990s. This study finds that the higher the PM_2.5_ concentration, the lower the willingness of the floating population to give birth to a second child, after controlling for the first child’s sex and age, and individual-, policy- and city-level factors. There exists significant regional disparity between PM_2.5_ concentrations and the second birth intentions of the floating population. Migrants living in south China (especially central–south and southwestern regions) are more sensitive to smog and thus have a lower level of resilience to air pollution and resultantly fewer intentions to have a second child, compared to the group coming from the north and still living in the north, who have a relatively higher tolerance to smog and thus more intentions for second births. China’s policy response to low fertility should, firstly, take environmental factors into account, and secondly, adopt effective methods and strategies to eliminate smog (air population broadly) not only in the urban areas of north China but also in the southern parts of the country. PM_2.5_ is a key air pollutant causing not only adverse human health problems but also a significant impact on birth intentions. Policies and practical schemes designed for alleviating smog hazards and for raising population fertility should factor in regional geospatial differences in PM_2.5_ concentration (or severity of smog) and the birth intentions of diverse groups of the floating population.

Two limitations of this study deserve mention. First, this research used only the 2014 floating population data to assess the impact of smog on fertility intentions. If large-scale data covering all residents can be collected in future studies, through longitudinal observations or quasi-experimental methods, a more comprehensive understanding of the causal effects of smog on fertility intentions (and fertility behavior) will become possible. Second, this study found that an increase in smog in the provinces of the north region (especially Hebei and Shaanxi) facilitates migrants’ birth intentions. This result signals out that apart from the northerners’ higher levels of resilience, other environmental and non-environmental factors might have mediated people’s birth intentions in China. More studies will be needed in this research field.

## Figures and Tables

**Table 1:** Descriptive summary of the sample.

Factors	Definition	South China (*N* = 18,603)	North China (*N* = 15,788)	All (*N* = 34,391)
Second birth intention	Yes (%)	22.83	19.47	21.29
Air pollution	PM_2.5_ concentration (10 μg/m^3^)	5.69	6.85	6.22
Demographic, socioeconomic factors	Sex			
	Male (%)	59.83	63.40	61.47
	Female (%)	40.17	36.60	38.53
	Household registration status			
	Rural (%)	81.37	79.64	80.57
	Urban (%)	18.63	20.36	19.43
	Age	33.77	34.45	34.08
	Schooling (years)	10.34	10.39	10.37
	Monthly income (100 yuan)	41.29	39.26	40.36
	Social status			
	Employee (%)	58.14	53.69	56.10
	Employer (%)	10.06	9.86	9.97
	Self-management (%)	30.44	34.54	32.32
	Others (%)	1.36	1.91	1.61
Second birth policy	Eligible couples (%)	13.07	12.66	12.88
	Knowing the policy (%)	94.08	92.71	93.45
First child	Sex			
	Boy (%)	62.87	60.21	61.65
	Girl (%)	37.13	39.39	38.35
	Age (years)	8.93	9.45	9.17
City level	Total GDP (hundred billion yuan)	7.505	6.659	7.117
	Per capita GDP (thousand yuan)	75.505	78.578	76.916
	Population density (100 people/km^2^)	7.816	4.77	6.42
	Percentage of the secondary industry (%)	47.55	45.62	46.66

Data source: The Chinese Floating Population Dynamic Survey, 2014. GDP, gross domestic product.

**Table 2:** Probit models: an estimate of second birth intentions of the floating population.

Variable	All Cities				40 Cities
	Model 1 Coef. (S.E.)	Model 2 Coef. (S.E.)	Model 3 Coef. (S.E.)	Model 4 Coef. (S.E.)	Model 5 Coef. (S.E.)
PM_2.5_ concentration (10 μg/m^3^)	−0.064 *** (0.007)	−0.058 *** (0.008)	−0.063 *** (0.008)	−0.061 *** (0.007)	−0.087 *** (0.012)
Female (male = 0)		−0.246 *** (0.028)	−0.162 *** (0.030)	−0.153 *** (0.030)	−0.153 *** (0.049)
Age		−0.092 *** (0.002)	−0.051 *** (0.004)	−0.050 *** (0.004)	−0.044 *** (0.007)
Schooling (years)		0.022 *** (0.005)	−0.014 ** (0.006)	−0.010 * (0.006)	−0.009 (0.009)
Urban hukou (rural = 0)		−0.230 *** (0.043)	−0.349 *** (0.047)	−0.337 *** (0.048)	−0.375 *** (0.080)
Employer (employee = 0)		0.179 *** (0.049)	0.205 *** (0.053)	0.195 *** (0.054)	0.247 *** (0.091)
Self-management (employee = 0)		0.116 *** (0.031)	0.136 *** (0.032)	0.115 *** (0.033)	0.144 *** (0.054)
Others (employee = 0)		−0.043 (0.144)	−0.041 (0.155)	−0.048 (0.159)	0.196 (0.256)
Monthly income (100 yuan)		0.0003 (0.0003)	−0.0003 (0.0003)	0.0001 (0.0003)	0.0002 (0.0005)
Eligible couples (no = 0)			0.578 *** (0.040)	0.594 *** (0.041)	0.616 *** (0.063)
Knowing the second–child policy (no = 0)			0.027 (0.055)	0.048 (0.055)	0.045 (0.093)
First child’s sex (boy = 0)			0.734 *** (0.029)	0.733 *** (0.029)	0.714 *** (0.045)
First child’s age			−0.051 *** (0.005)	−0.053 *** (0.005)	−0.060 *** (0.008)
Total GDP (100 billion yuan)				−0.012 *** (0.003)	−0.002 (0.005)
Per capita GDP (thousand yuan)				−0.001 ** (0.0006)	−0.002 * (0.001)
Population density (100 people/km^2^)				0.002 (0.003)	−0.0007 (0.005)
Secondary industry (%)				−0.0005 (0.002)	0.0028 (0.003)
Constant	−0.487 *** (0.045)	2.441 *** (0.109)	1.474 *** (0.132)	1.588 *** (0.156)	1.364 *** (0.288)
Observations (N)	34,391	34,391	34,391	34,391	14,368
Wald Chi^2^ statistics	84.10	2061.03	2104.24	2150.42	865.71
Pseudo R^2^	0.006	0.168	0.207	0.211	0.210
% of correct prediction	78.75	78.71	80.45	80.56	82.17

Note: *** *p* < 0.01; ** *p* < 0.05; * *p* < 0.1. Numbers in brackets are robust standard errors.

**Table 3:** Differences in the influence of smog on the second birth intention of the floating population across individual-level variables.

Variable	Model 6 Coef. (S.E.)	Model 7 Coef. (S.E.)	Model 8 Coef. (S.E.)	Model 9 Coef. (S.E.)	Model 10 Coef. (S.E.)	Model 11 Coef. (S.E.)	Model 12 Coef. (S.E.)
PM_2.5_ concentration (10 μg/m^3^)	−0.063 *** (0.009)	−0.044 *** (0.012)	−0.051 *** (0.009)	−0.059 *** (0.007)	−0.058 *** (0.007)	−0.047 *** (0.010)	−0.051 *** (0.010)
Interaction terms							
Sex × PM_2.5_	0.009						
	(0.017)						
Old age × PM_2.5_		−0.024					
		(0.017)					
Middle age × PM_2.5_		−0.013					
		(0.036)					
Senior high school × PM_2.5_			−0.025				
			(0.021)				
University degree × PM_2.5_			−0.009				
			(0.021)				
Non-rural hukou × PM_2.5_				0.004			
				(0.024)			
Eligible couples × PM_2.5_					−0.005		
					(0.025)		
Have a girl × PM_2.5_						−0.025	
						(0.016)	
Monthly income of 3000–4000 × PM_2.5_							−0.019
							(0.018)
Monthly income of 5000–7000 × PM_2.5_							−0.020
							(0.029)
Monthly income > 7000 × PM_2.5_							0.005
							(0.040)
Other variables	Control	Control	Control	Control	Control	Control	Control
Observations (N)	34,391	34,391	34,391	34,391	34,391	34,391	34,391

Note: *** *p* < 0.01; ** *p* < 0.05; * *p* < 0.1. The reproductive age was categorized into three groups: young age: <30 years old (reference group); middle age: 30–39 years; old age: 40–49 years. Monthly income was grouped into four scales: <3000 yuan (reference group); 3000–4000 yuan; 5000–7000 yuan; >7000 yuan. The results for other independent variables in regression are not included in the table due to word limit. Such variables include age, sex, education, social class, hukou status, income, the first child’s sex and age, eligibility for a second child, knowing the second-child policy or not, total GDP and per capita GDP in a city, urban population density, and proportion of the secondary industry.

**Table 4:** Probit models: estimation of second birth intention of the floating population in south and north China.

Variable	Living in South China		Living in North China	
	Originating from the South	Originating from the North	Originating from the South	Originating from the North
	Model 13 Coef. (S.E.)	Model 14 Coef. (S.E.)	Model 15 Coef. (S.E.)	Model 16 Coef. (S.E.)
PM_2.5_ concentration (10 μg/m^3^)	−0.130 *** (0.012)	−0.171 *** (0.042)	0.042 (0.034)	0.049 *** (0.012)
Female (male = 0)	−0.135 *** (0.039)	−0.335 *** (0.101)	−0.280 *** (0.105)	−0.156 *** (0.043)
Age	−0.043 *** (0.005)	−0.079 *** (0.014)	−0.066 *** (0.014)	−0.070 *** (0.006)
Schooling (years)	−0.015 * (0.008)	0.019 (0.020)	−0.007 (0.020)	−0.010 (0.008)
Non-rural (rural = 0)	−0.334 *** (0.064)	−0.274 * (0.159)	−0.144 (0.146)	−0.356 *** (0.067)
Employer (employee = 0)	0.211 *** (0.071)	0.309 (0.205)	0.155 (0.151)	0.168 ** (0.079)
Self-management (employee = 0)	0.068 (0.043)	0.333 *** (0.125)	0.330 *** (0.118)	0.174 *** (0.049)
Others (employee = 0)	−0.169 (0.168)	0.559 (0.527)	0.149 (0.422)	−0.179 (0.143)
Monthly income (hundred yuan)	0.0003 (0.0004)	−0.0030 * (0.0017)	0.0006 (0.0006)	0.0006 (0.0006)
Eligible couples (no = 0)	0.632 *** (0.055)	0.753 *** (0.137)	0.716 *** (0.133)	0.525 *** (0.060)
Knowing the policy (no = 0)	0.035 (0.072)	−0.099 (0.195)	0.501 ** (0.216)	0.040 (0.076)
First child’s sex (boy = 0)	0.748 *** (0.038)	0.929 *** (0.096)	0.573 *** (0.101)	0.649 *** (0.042)
First child’s age	−0.062 *** (0.006)	−0.031 * (0.016)	−0.033 ** (0.015)	−0.017 ** (0.008)
Total GDP (hundred billion yuan)	−0.015 *** (0.004)	0.018 (0.014)	−0.041 *** (0.015)	−0.016 *** (0.005)
Per capita GDP (thousand yuan)	−0.001 (0.001)	−0.002 (0.002)	0.0003 (0.003)	0.002 ** (0.001)
Population density (100 people/km^2^)	−0.001 (0.005)	−0.022 (0.014)	0.061 ** (0.025)	0.018 * (0.010)
Secondary industry (%)	0.002 (0.002)	0.003 (0.009)	−0.014 *** (0.005)	0.002 (0.002)
Intercept	1.772 *** (0.211)	2.747 *** (0.643)	1.066 ** (0.540)	0.865 *** (0.229)
Observations (N)	16,887	1716	2117	13,671
Wald Chi^2^ statistics	1407.63	293.45	226.49	687.09
Pseudo R^2^	0.227	0.250	0.219	0.174
% of correct prediction	80.92	76.59	81.71	81.89

Note: * *p* < 0.05; ** *p* < 0.01; *** *p* < 0.001. Numbers in brackets are robust standard errors.

**Table 5:** The impact of smog on second birth intentions of the floating population in seven regions of China.

Regions	North	Northeast	Northwest	Yangtze River Delta	Central–South	South	Southwest
PM_2.5_ concentration (10 μg/m^3^)	0.029 ** (0.013)	−0.084 (0.058)	−0.057 (0.045)	−0.018 (0.034)	−0.137 *** (0.020)	−0.037 (0.040)	−0.278 *** (0.075)
Observations (N)	7671	3717	4400	6597	4504	4375	3127

Note: *** *p* < 0.01; ** *p* < 0.05. Numbers in brackets are robust standard errors. The results for other independent variables in regression are not included in the table due to the word limit. Such variables include age, sex, education, social class, hukou status, income, the first child’s sex and age, eligibility for a second child, knowing the second-child policy or not, total GDP and per capita GDP in a city, urban population density, and proportion of the secondary industry.

**Table 6:** The impact of smog on the second birth intention of the floating population in major provinces in China.

**Provinces**	**Hebei**	**Shanxi**	**Shandong**	**Henan**	**Inner Mongolia**
PM_2.5_ concentration (10 μg/m^3^)	0.214 *** (0.081)	−0.012 (0.088)	−0.012 (0.035)	−0.041 (0.125)	−0.191 *** (0.103)
Observations	819	773	2303	674	1119
**Provinces**	**Heilongjiang**	**Jilin**	**Liaoning**	**Shaanxi**	**Gansu**
PM_2.5_ concentration (10 μg/m^3^)	−0.169 (0.268)	−0.352 (0.301)	−0.012 (0.133)	0.141 * (0.083)	−0.517 *** (0.192)
Observations	1861	741	1049	1203	828
**Provinces**	**Jiangsu**	**Zhejiang**	**Anhui**	**Hubei**	**Hunan**
PM_2.5_ concentration (10 μg/m^3^)	−0.447 *** (0.173)	0.033 (0.042)	0.009 (0.133)	−0.279 *** (0.073)	−0.199 *** (0.052)
Observations	2711	2211	1624	1347	1140
**Provinces**	**Guangdong**	**Guizhou**	**Fujian**	**Sichuan**	**Yunnan**
PM_2.5_ concentration (10 μg/m^3^)	−0.119 * (0.094)	−0.476 ** (0.258)	−0.548 (0.383)	0.623 (0.496)	0.301 (0.240)
Observations	1836	500	1337	920	457

Note: *** *p* < 0.01; ** *p* < 0.05; * *p* < 0.1. Numbers in brackets are robust standard errors. The results for other independent variables in regression are not included in the table due to the word limit. Such variables include age, sex, education, social class, hukou status, income, the first child’s sex and age, eligibility for a second child, knowing the second-child policy or not, total GDP and per capita GDP in a city, urban population density, and proportion of the secondary industry.

## References

[1] Chen W., Wu L.L. (2006). Research on the relationships between migration and fertility in China (In Chinese). Popul. Res..

[2] National Bureau of Statistics of China (2015). China Statistical Yearbook 2014.

[3] Liang Z., Li Z., Ma Z. (2014). Changing patterns of the floating population in China, 2000–2010. Popul. Dev. Rev..

[4] National Health and Family Planning Commission (2017). China’s Health and Family Planning Statistical Yearbook 2016.

[5] Du M. (2013). Change trend, social integration and the innovation of management system of floating population in China. Public Manag..

[6] Feyisetan B., Casterline J.B. (2000). Fertility preferences and contraceptive change in developing countries. Int. Fam. Plan. Perspect..

[7] Wang Z., Zhang F., Wu F. (2016). Intergroup neighbouring in urban China: Implications for the social integration of migrants. Urban Stud..

[8] Guo Z., Wu Z., Schimmele C.M., Li S. (2012). The effect of urbanization on China’s fertility. Popul. Res. Policy Rev..

[9] Feeney G., Wang F. (1993). Parity progression and birth intervals in China: The influence of policy in hastening fertility decline. Popul. Dev. Rev..

[10] Goldstein J.R., Sobotka T., Jasilioniene A. (2009). The end of “lowest-low” fertility?. Popul. Dev. Rev..

[11] EPI (2014). Environmental Performance Index 2014.

[12] Zhou M., He G., Fan M., Wang Z., Liu Y., Ma J., Ma Z., Liu J., Liu Y., Wang L. (2015). Smog episodes, fine particulate pollution and mortality in China. Environ. Res..

[13] EPA (2009). Integrated Science Assessment for Particulate Matter.

[14] Li P., Xin J., Wang Y., Wang S., Li G., Pan X., Liu Z., Wang L. (2013). The acute effects of fine particles on respiratory mortality and morbidity in Beijing, 2004–2009. Environ. Sci. Pollut. Res..

[15] Lim S.S., Vos T., Flaxman A.D., Danaei G., Shibuya K., Adair-Rohani H., Aryee M. (2012). A comparative risk assessment of burden of disease and injury attributable to 67 risk factors and risk factor clusters in 21 regions, 1990–2010: A systematic analysis for the Global Burden of Disease Study 2010. Lancet.

[16] Lin H., Tao J., Kan H., Qian Z., Chen A., Du Y., Ma W. (2018). Ambient particulate matter air pollution associated with acute respiratory distress syndrome in Guangzhou, China. J. Expo. Sci. Environ. Epidemiol..

[17] Dadvand P., Parker J., Bell M.L., Bonzini M., Brauer M., Darrow L.A., Gehring U., Glinianaia S.V., Gouveia N., Ha E.H. (2013). Maternal Exposure to Particulate Air Pollution and Term Birth Weight: A Multi-Country Evaluation of Effect and Heterogeneity. Environ. Health Perspect..

[18] Rich D.Q., Liu K., Zhang J., Thurston S.W., Stevens T.P., Pan Y., Kane C., Weinberger B., Ohman-Strickland P., Woodruff T. (2015). Differences in birth weight associated with the 2008 Beijing Olympics air pollution reduction: Results from a natural experiment. Environ. Health Perspect..

[19] Carré J., Gatimel N., Moreau J., Parinaud J., Léandri R. (2017). Does air pollution play a role in infertility? A systematic review. Environ. Health.

[20] Ha S., Sundaram R., Louis G.M.B., Nobles C., Seeni I., Sherman S., Mendola P. (2018). Ambient air pollution and the risk of pregnancy loss: A prospective cohort study. Fertil. Steril..

[21] Vizcaíno M.A.C., González-Comadran M., Jacquemin B. (2016). Outdoor air pollution and human infertility: A systematic review. Fertil. Steril..

[22] Sun C., Scharping T. (1997). Floating population in Shanghai: A perspective on social transformation in China. Floating Population and Migration in China: The Impact of Economic Reforms.

[23] Schoen R., Astone N.M., Kim Y.J., Nathanson C.A., Fields J.M. (1999). Do fertility intentions affect fertility behavior?. J. Marriage Fam..

[24] Testa M.R. (2014). On the positive correlation between education and fertility intentions in Europe: Individual-and country-level evidence. Adv. Life Course Res..

[25] Wu Y., Yang Y.Y., Wei X.J., Chen E. (2017). What determines “giving birth to a son”: The social transformation of how institution and culture affect women’s fertility choices. J. Chin. Sociol..

[26] Zeng Y., Hesketh T. (2016). The effects of China’s universal two-child policy. Lancet.

[27] Guo W. (2017). The changes of disability-free life expectancy and inter-generation support for the elderly in China: 2005–2010. Cross-Cultural and Cross-Disciplinary Perspectives in Social Gerontology.

[28] Wang F., Gu B., Cai Y. (2016). The End of China’s One-Child Policy. Stud. Fam. Plan..

[29] Bao L., Chen F., Zheng Z. (2017). Transition in second birth intention in a low fertility context: The case of Jiangsu, China. Asian Popul. Stud..

[30] Qin Y., Wang F. (2017). Too early or too late: What have we learned from the 30-year two-child policy experiment in Yicheng, China?. Demogr. Res..

[31] Yang J.H. (2018). Research on Migrants’ Fertility Intention of the Second Child (In Chinese). Chin. J. Popul. Sci..

[32] Zhang J. (2017). The evolution of China’s one-child policy and its effects on family outcomes. J. Econ. Perspect..

[33] Piotrowski M., Tong Y. (2016). Education and fertility decline in China during transitional times: A cohort approach. Soc. Sci. Res..

[34] Becker G.S., Lewis H.G. (1973). On the Interaction between the Quantity and Quality of Children. J. Political Econ..

[35] Cai Y. (2010). China’s below-replacement fertility: Government policy or socioeconomic development?. Popul. Dev. Rev..

[36] Bongaarts J. (1997). Trends in unwanted childbearing in the developing world. Stud. Fam. Plan..

[37] Bongaarts J. (2003). Completing the fertility transition in the developing world: The role of educational differences and fertility preferences. Popul. Stud..

[38] Gu P., Ma X. (2013). Investigation and analysis of a floating population’s settlement intention and environmental concerns: A case study in the Shawan River Basin in Shenzhen. China. Habitat Int..

[39] Démurger S., Gurgand M., Li S., Yue X. (2009). Migrants as second-class workers in urban China? A decomposition analysis. J. Comp. Econ..

[40] Fang H., Eggleston K.N., Rizzo J.A., Zeckhauser R., John F. (2010). Female Employment and Fertility in Rural China.

[41] Moav O. (2005). Cheap children and the persistence of poverty. Econ. J..

[42] Engelhardt H., Kögel T., Prskawetz A. (2004). Fertility and women’s employment reconsidered: A macro-level time-series analysis for developed countries, 1960–2000. Popul. Stud..

[43] Zhou M., Guo W. (2019). Fertility intentions of having a second child among the floating population in China: Effects of socioeconomic factors and home ownership. Popul. Space Place.

[44] Martin E. (1990). The woman in the body: A cultural analysis of reproduction. Birth.

[45] Poston D.L., Gu B., Liu P.P., McDaniel T. (1997). Son preference and the sex ratio at birth in China: A provincial level analysis. Soc. Biol..

[46] Poston D.L. (2002). Son preference and fertility in China. J. Biosoc. Sci..

[47] Attané I., Guilmoto C.Z. (2007). Watering the Neighbours’ Garden: The Growing Female Demographic Deficit in Asia.

[48] Mohabir N., Jiang Y., Ma R. (2017). Chinese floating migrants: Rural-urban migrant laborers’ intentions to stay or return. Habitat Int..

[49] Cheng T., Selden M. (1994). The origins and social consequences of China’s hukou system. China Q..

[50] Chan K.W. (2010). The household registration system and migrant labor in China: Notes on a debate. Popul. Dev. Rev..

[51] Wong K., Fu D., Li C.Y., Song H.X. (2007). Rural migrant workers in urban China: Living a marginalised life. Int. J. Soc. Welf..

[52] Lindstrom D.P., Saucedo S.G. (2002). The short-and long-term effects of US migration experience on Mexican women’s fertility. Soc. Forces.

[53] Wolf K., Mulder C.H. (2018). Comparing the fertility of Ghanaian migrants in Europe with non-migrants in Ghana. Popul. Space Place.

[54] Goldstein A., White M. (1997). Migration, fertility, and state policy in Hubei Province, China. Demography.

[55] Massey D.S. (2004). Segregation and stratification: A biosocial perspective. Du Bois Rev..

[56] Xie Y.B., Chen J., Li W. (2014). An assessment of PM2.5 related health risks and impaired values of Beijing residents in a consecutive high-level exposure during heavy haze days. Huan Jing Kexue.

[57] Ma Y., Chen R., Pan G., Xu X., Song W., Chen B., Kan H. (2011). Fine particulate air pollution and daily mortality in Shenyang, China. Sci. Total Environ..

[58] Wong C.M., Lai H.K., Tsang H., Thach T.Q., Thomas G.N., Lam K.B., Chan K.P., Yang L., Lau A.K., Ayres J.G. (2015). Satellite-based estimates of long-term exposure to fine particles and association with mortality in elderly Hong Kong residents. Environ. Health Perspect..

[59] Grant W. (1996). Autos, Smog and Pollution Control. The Politics of Air Quality Management in California.

[60] Kahn M.E. (2000). Smog reduction’s impact on California county growth. J. Reg. Sci..

[61] Nieuwenhuijsen M.J., Basagaña X., Dadvand P., Martinez D., Cirach M., Beelen R., Jacquemin B. (2014). Air pollution and human fertility rates. Environ. Int..

[62] Perera F.P., Tang D., Wang S., Vishnevetsky J., Zhang B., Diaz D., Rauh V. (2012). Prenatal polycyclic aromatic hydrocarbon (PAH) exposure and child behavior at age 6–7 years. Environ. Health Perspect..

[63] Zhang H., Wang Z., Zhang W. (2016). Exploring spatiotemporal patterns of PM_2.5_ in China based on ground-level observations for 190 cities. Environ. Pollut..

[64] Zhang Z., Wang W., Cheng M., Liu S., Xu J., He Y., Meng F. (2017). The contribution of residential coal combustion to PM2. 5 pollutions over China’s Beijing-Tianjin-Hebei region in winter. Atmos. Environ..

[65] Greene W. (2008). Econometric Analysis.

[66] Jiang Q., Li Y., Sánchez-Barricarte J.J. (2016). Fertility intention, son preference, and second childbirth: Survey findings from Shaanxi Province of China. Soc. Indic. Res..

[67] Zheng Z.Z., Cai Y., Wang F., Gu B.C. (2009). Below-replacement fertility and childbearing intention in Jiangsu Province, China. Asian Popul. Stud..

[68] Hicken M.T., Lee H., Morenoff J., House J.S., Williams D.R. (2014). Racial/ethnic disparities in hypertension prevalence: Reconsidering the role of chronic stress. Am. J. Public Health.

[69] Xu P., Chen Y., Ye X. (2013). Haze, air pollution, and health in China. Lancet.

